# The role of the transsulfuration pathway in spermatogenesis of vitamin D deficient mice

**DOI:** 10.1038/s41598-023-45986-4

**Published:** 2023-11-06

**Authors:** Narges Jamshidian-Ghalehsefidi, Farzaneh Rabiee, Marziyeh Tavalaee, Shaghayegh Kiani, Farnaz Pouriayevali, Mazdak Razi, Maurizio Dattilo, Mohammad Hossein Nasr-Esfahani

**Affiliations:** 1 ACECR Institute of Higher Education, Isfahan Branch, Isfahan, Iran; 2 Department of Animal Biotechnology, Reproductive Biomedicine Research Center, Royan Institute for Biotechnology, ACECR, Isfahan, Iran; 3 Department of Animal Biotechnology, Cell Science Research Center, Royan Institute for Biotechnology, ACECR, Isfahan, Iran; 4 Division of Histology and Embryology, Department of Basic Sciences, Faculty of Veterinary Medicine, Urmia University, Urmia, Iran; 5 R&D Department, Parthenogen, Lugano, Switzerland

**Keywords:** Cell biology, Physiology

## Abstract

Vitamin D deficiency is a global health problem and has been linked to defective spermatogenesis and male infertility. In this study, we aimed to investigate the main enzymes involved in the transsulfuration pathway of 1-carbon metabolism, and spermatogenesis function. Therefore, sixteen male C57 mice were addressed to a control (standard diet) or vitamin D deficient (VDD) diet for 14 weeks. The results show that compared to the standard diet, VDD increased final body weight and reduced sperm quality, caused damage to the testicular structure, and decreased the serum levels of testosterone. In addition, serum concentrations of homocysteine, vitamin B12, and sperm oxidative stress markers increased. In testicular tissues, the CBS and CSE protein levels were down-regulated whereas HO-1 was up-regulated at both mRNA and protein expression levels. Within a mice deprivation model, VDD deeply suppressed testosterone and impaired spermatogenesis with oxidative stress-mediated mechanisms. The effects of the deprivation appeared to be at least in part independent of genomic and receptor-mediated vitamin D actions and suggest a specific impairment of the alternative transsulfuration pathway.

## Introduction

Vitamin D is a group of fat-soluble steroids playing a well-established role in adjusting calcium and phosphorus homeostasis and stimulating the mineralization of bones^1,2^. The main compounds in this steroid group are cholecalciferol (vitamin D3) and ergocalciferol (vitamin D2)^2^. Human vitamin D deficiency (vitamin D < 20 ng/ml) is increasingly recognized as a public health problem, but the health implications are not yet adequately understood^3^. Vitamin D biological actions are mainly mediated via the active form of vitamin D3 binding to the vitamin D receptors (VDRs) in the target tissues. VDRs and the vitamin D metabolizing enzymes (CYP2R1, CYP27B1, and CYP24A1) are expressed in germ cells, spermatozoa, Leydig and epithelial cells lining the male reproductive system^4–6^.

A novel and non-canonical pathway for activating vitamin D3 begins with CYP11A1^7^, introducing hydroxyl groups at C20 or C22, followed by subsequent hydroxylations at C20, C22, C23, and/or C17. These compounds can be selectively hydroxylated by enzymes like CYP27A1, CYP24A1, CYP2R1, and CYP3A4. Except for C17 hydroxylated intermediates, most can undergo further hydroxylation at C1a. These intermediates can be produced in skin cells, placenta, and pig adrenal glands^8^.

Notably, CYP11A1 catalyzes the hydroxylation of vitamin D2, resulting in the formation of 20(OH)D2, 17,20(OH)_2_D2, and 17,20,24(OH)_3_D2. Additionally, CYP27B1 converts 20(OH)D3 into 1,20(OH)_2_D2. These vitamin D2 metabolites are also generated in the human placenta, skin cells, and the adrenal glands of pigs^9^. CYP11A1 is highly expressed in key steroid-producing tissues like the adrenal cortex, testes, ovaries, and placenta, well-documented^10^.

The hydroxyderivatives derived from CYP11A1 are characterized as non-calcemic or having low calcemic properties^8^. Similar to 1,25(OH)_2_D3, they have the ability to modify gene expression by binding to the genomic site of the VDR. Furthermore, these vitamin D3 hydroxyderivatives derived from CYP11A1 can also bind to other nuclear receptors, such as the aryl hydrocarbon receptor (AhR), retinoic acid orphan receptors (ROR) α and γ, and liver X receptor (LXR)α and β, thereby influencing their expression and activities.

Nevertheless, it's important to keep in mind the enzymatic activation of vitamin D3 or sterol precursors through receptor-independent pathways. Notably, hydroxymetabolites of vitamin D3 produced by CYP11A1, including 20(OH)D3 and 20,23(OH)_2_D3, have demonstrated anti-inflammatory and anti-oxidative effects, similar to the effects observed with the classical active form of vitamin D3, 1,25(OH)2D3^11^. Accordingly, vitamin D is likely to play a significant role in spermatogenesis and sperm quality in humans and animals^12^.

Vitamin D deficiency (VDD) is associated with increased oxidative stress (OS) in a variety of clinical conditions including obesity^13^, diabetes in the elders^14^, and severe asthma^15^ and this may be true also in male infertility. A study on infertile men candidates for intra-cytoplasmic sperm injection (ICSI) confirmed the association between the amount of vitamin D in their diet and OS and the DNA integrity of their sperm^16^. These observations prompted us to use an animal model to investigate the mechanistic connection between VDD and these perturbations. The most common strategy to induce VDD in animal models is using a vitamin D deficient diet^17,18^ that can be backed by VDR^19^ or 1-alpha hydroxylase^20^ knockout models. Another method is inducing simultaneous vitamin D and calcitriol deficiency by feeding the animals with a vitamin D deficient diet but high calcium and phosphorous, and subsequently administering the paricalcitol as a calcitriol analogue (32 ng/day)^21,22^. Herein, the dietary approach was used to achieve VDD, while keeping a normal serum calcium level.

OS should be balanced by the endogenous antioxidant system, where the reducing agent is glutathione (GSH). De-novo synthesis of GSH occurs via the transsulfuration pathway that converts homocysteine (Hcy), generated in the transmethylation reactions, to cysteine, which is then used by glutathione synthase (GS) to produce GSH^23^. According to the canonical trans-sulfuration pathway, the first enzyme, cystathionine-β‐synthase (CBS), binds Hcy to serine to generate cystathionine. A second enzyme, cystathionine-γ‐lyase (CSE), produces cysteine from cystathionine. Cysteine is the mandatory substrate for the synthesis of proteins, of GSH, and of taurine^24^. However, the same enzymes exert an ambiguous recognition of their substrates and may act in a reverse manner, called alternative transsulfurations, using cysteine, Hcy, and cystathionine as substrates for the generation of the gasotransmitter hydrogen sulfide (H_2_S). This is a reducing substance that mainly acts by binding the heme center of metalloproteins or by persulfidation of protein cysteines (Protein-S-SH), which changes their activity^25^. In addition, H_2_S easily enters mitochondria where it can be used as a source of reducing equivalents for the synthesis of ATP^26^. In summary, transsulfurations can either feed GSH generation and antioxidant defenses or H_2_S release according to the direction of the reactions.

The signals leading CBS and CSE to work in their reverse manner are matter of research, however it was shown that another gasotransmitter, carbon monoxide (CO), is able to activate the pathway by binding the heme center of CBS^27^. Upon CO binding, CBS changes its activity and CSE follows, likely due to changed concentrations of substrates. CO is the product of heme oxygenase (HO), mainly the inducible heme oxygenase (HO-1) coded by the gene *HMOX1*, that catabolizes the heme group to produce ferrous iron, biliverdin and CO. HO-1 is known to be a stress response protein possessing important cytoprotective, antioxidant, and anti-inflammatory properties^28^. In addition, based on Kabil et al.^27^, HO-1 should be considered as a main modulator of transsulfuration.

The cysteine-producing (canonical reactions) or H_2_S-releasing (alternative reactions) enzymes, CBS and CSE, are proven to occur in mouse testes^29^ and human spermatozoa^30^. Numerous studies have demonstrated that one-carbon metabolism dysfunction, particularly the dysfunction of the transsulfuration enzymes CBS and CSE, plays a role in male infertility^31,32^, and also a specific deficit in the H_2_S output is reported^30^. In the sperm, the persulfidation of proteins, including glyceraldehyde-3-phosphate dehydrogenase (GAPDH), tubulin, and anchor protein A-kinase, as a part of the redox signaling pathway, is strongly controlled via enzymatic H_2_S formation and is needed for sperm viability^33^.

Whatever the direction of transsulfuration reactions, the gene coding for the regulating enzyme of the pathway, *CBS*, carries Vitamin D Response elements (VDREs) and is responsive to vitamin D^34^. Thus, its expression is likely modified by vitamin D deprivation, which might account for the OS associated to VDD.

In summary, VDD is likely to hamper the fertility function and a perturbation of the transsulfuration pathway is likely involved. However, few data are available on the effect of VDD on the transsulfuration pathway and, so far, there have been only scant reports about the expression and role of HO-1 in testis. The present study was intended to assess the effects of VDD on sperm functions and enzymes involved in transsulfuration pathway as well as HO-1 expression in a mouse model.

## Results

### Effect of vitamin D deficiency on body weight and testicular weight and histology

**Figure 1:**
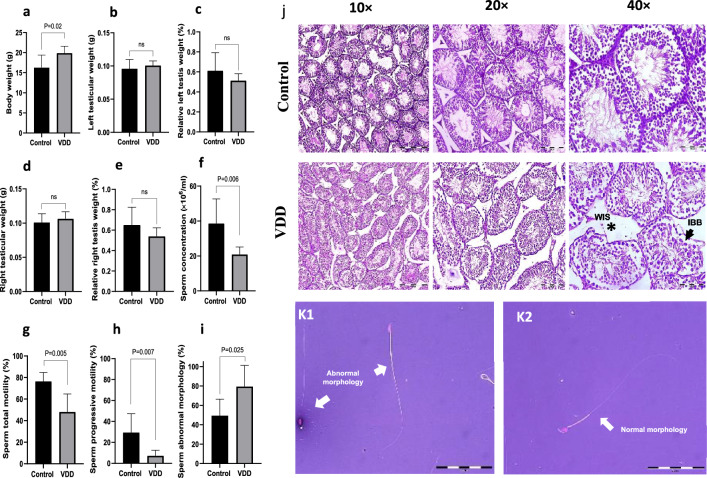
Comparison between control and VDD groups of (**a**) body weight, (**b, c**) left testicular weight, (**d, e**) right testicular weight, (**f**) sperm concentration, (**g**) sperm total motility, (**h**) sperm progressive motility, and (**i, k**) sperm abnormal morphology. (**j**) Histological testicular structure. *WIS* wide interstitial space, the arrow, *IBB* irregular basement membranes. Data are presented as mean ± SD and analyzed by independent-samples T-test. *P* < 0.05. *VDD* vitamin D deficiency (N = 8).

The mice final body weight was significantly (*P* = 0.02) increased in the VDD group (19.88 ± 1.71) compared to the control group (16.28 ± 3.11). Notably, there was no significant difference observed in the absolute and relative testicular weights of the mice between the two groups (Fig. 1a–e). The testicular sections of control mice revealed normal architecture (Fig. 1j) while images of the testes of the VDD group (Fig. 1j) showed histopathological alterations such as increased irregular basement membranes, wide interstitial space, enhanced cell debris in the tubular lumen, the presence of vacuolar structures, and germ cells loss.

### Effect of vitamin D deficiency on sperm parameters

As depicted in Fig. 1f–i, VDD significantly reduced sperm concentration (38.6 ± 14.1 versus 20.85 ± 4.27, *P* = 0.006), total motility (48.08 ± 16.65 versus 76.47 ± 8.20, *P* = 0.005) and progressive motility (29.37 ± 17.95 versus 7.21 ± 5.36, *P* = 0.007). In addition, VDD significantly increased sperm abnormal morphology compared to the controls (79.37 ± 21.86 versus 49.40 ± 17.06, *P* = 0.025, Fig. 1k).

### Effects of vitamin D deficiency on sperm oxidative status

**Figure 2:**
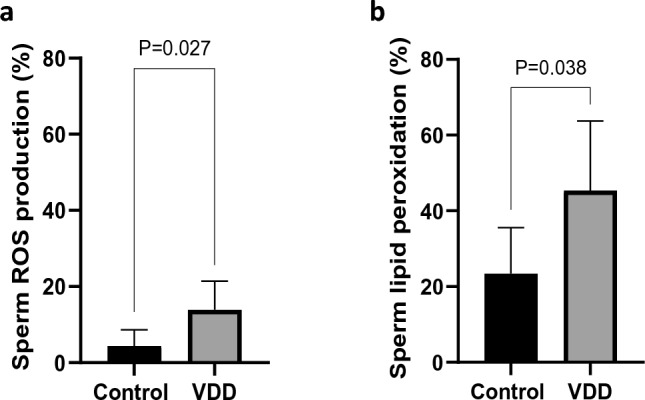
Comparison of sperm oxidative status including (**a**) sperm intracellular ROS production and (**b**) sperm lipid peroxidation between the control and VDD groups. Data are presented as mean ± SD and analyzed by independent-samples T-test. *P* < 0.05. *VDD* vitamin D deficiency (N = 8).

VDD induced a significant increase in oxidative stress (OS) markers (Fig. 2a, b). In the VDD group, both the production of ROS (13.87 ± 7.54 vs 4.40 ± 4.22, *P* = 0.027) and the sperm LPO level (45.37 ± 18.30 vs 23.40 ± 12.18, *P* = 0.038) increased significantly as compared to controls.

### Effects of vitamin D deficiency on sperm chromatin status

**Figure 3:**
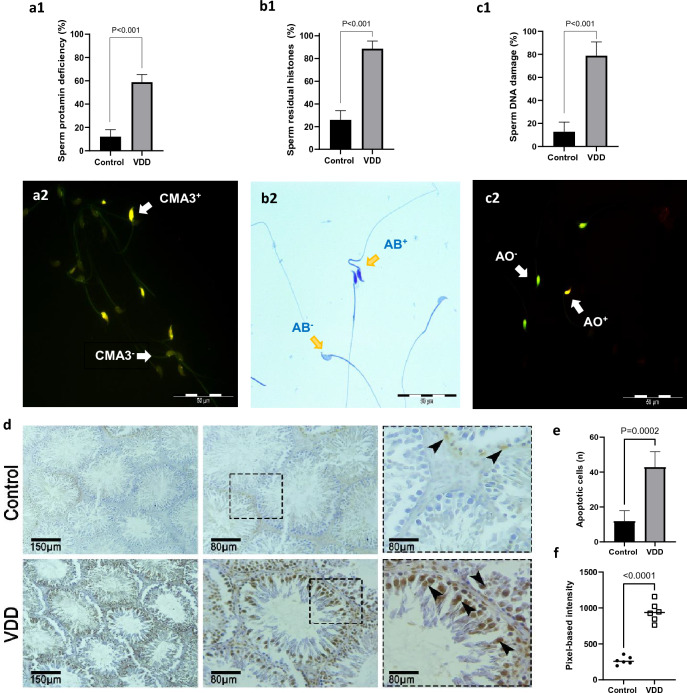
Comparison of sperm functional parameters including **(a1**, **2)** rate of sperms with an excess of protamine deficiency—Chromomycin A3 (CMA3) staining, (**b1, 2**) rate of sperms with an excess of residual histones—Aniline blue (AB) staining, (**c1**, **2**) sperm DNA damage—Acridine orange (AO) staining, and (**d–f**) mean percentage of TUNEL positive cells in testes cross-sections between the control and VDD groups. Data are presented as mean ± SD and analyzed by independent-samples T-test. *P* < 0.05. *VDD* vitamin D deficiency (N = 8).

The harmful effect of VDD on sperm chromatin and DNA status is depicted in Fig. 3a–c. In the VDD group the mean percentage of sperm chromatin protamine deficiency (58.75 ± 6.63 vs 12.00 ± 6.04, *P* < 0.001), and the percentage of sperm with excessive residual histones (88.62 ± 6.76 vs 26.0 ± 8.12, *P* < 0.001) were remarkably increased compared to the control group. In addition, the sperm DNA damage increased significantly in mice with VDD (78.87 ± 11.93) compared to the controls (12.80 ± 8.32, *P* < 0.001).

### Effects of vitamin D deficiency on testicular cells apoptosis

In this study, the TUNEL assay was used to evaluate apoptotic markers in testicular tissue sections. The results depicted in Fig. 3d–f indicate that the VDD group exhibited a higher number of apoptotic cells (42.80 ± 8.92 vs 11.80 ± 6.01, *P* = 0.0002) and intensity (943.7 ± 137.9 vs 268.7 ± 56.98, *P* < 0.0001) compared to the control group, indicating significant apoptotic damage.

### Effects of vitamin D deficiency on serum metabolites

**Figure 4:**
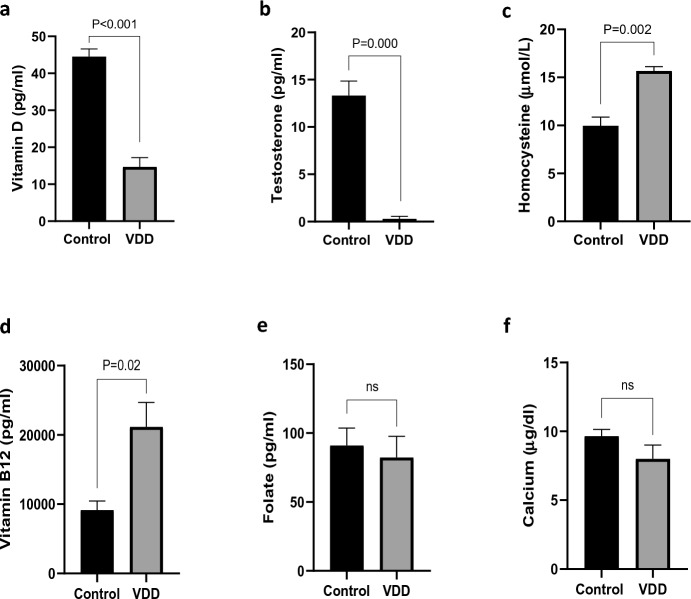
Comparison of serum levels of (**a**) vitamin D, (**b**) testosterone, (**c**) homocysteine, (**d**) vitamin B12, (**e**) folate, and (**f**) calcium between the control and VDD groups. Data are presented as mean ± SD and analyzed by independent-samples T-test. *P* < 0.05. *VDD* vitamin D deficiency (N = 3).

Our diet was effective in inducing a shortage of vitamin D (14.66 ± 2.51 pg/ml) as compared to the control diet (44.50 ± 2.12 pg/ml, *P* < 0.001, Fig. 4a). VDD significantly decreased testosterone level (0.29 ± 0.26 vs 13.33 ± 1.52 pg/ml, *P* < 0.001, Fig. 4b) whereas it significantly increased serum homocysteine level (15.66 ± 0.46 vs 9.95 ± 0.91 micromol/L, *P* = 0.002, Fig. 4c) and serum vitamin B12 level (21,170 ± 3516.1 vs 9168.5 ± 1301.2 pg/ml, *P* = 0.02, Fig. 4d). There were no significant differences in serum folate and calcium levels of mice in the VDD group in comparison with the control group (Fig. 4e, f), which confirms that the overfeeding with calcium and phosphorus (see methods) was effective in preventing derangements in calcium and phosphorus homeostasis.

### Effects of vitamin D deficiency on CBS, CSE, and HO-1 expression levels

**Figure 5:**
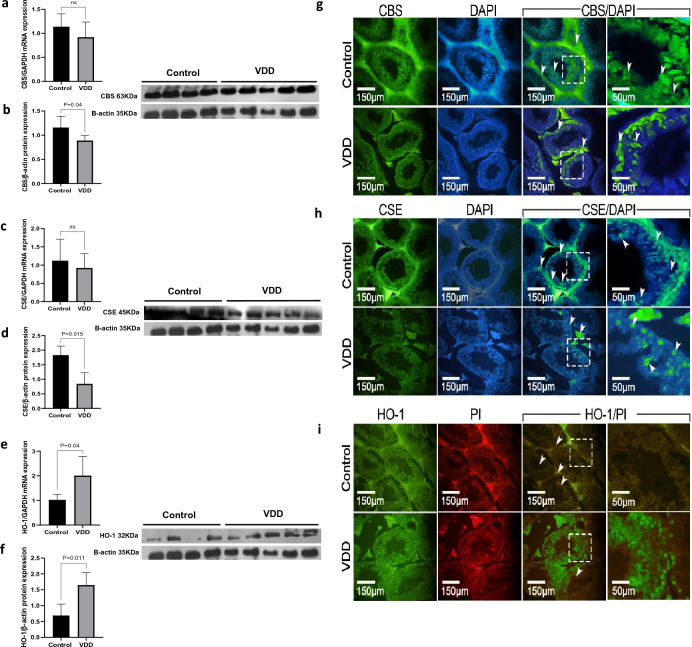
RT-PCR and western blot of H_2_S-releasing enzymes CBS, CSE, and HO-1 of mouse testis (**a–f**) in the control and VDD groups. Immunofluorescence of H_2_S-releasing enzymes CBS, CSE, and HO-1 (**g–i**) of testis sections. The immunofluorescence findings revealed decreased immunoreactivity of CBS and CSE in the testicular germ cells and spermatozoa of VDD-treated mice compared to the control group. Conversely, a notable increase in immunoreactivity for HO-1 was observed in the VDD group when compared to the control group. Data are presented as mean ± SD and analyzed by independent-samples T-test. *P* < 0.05. *CBS* cystathionine β-synthase, *CSE* cystathionine γ-lyase, *HO-1* heme oxygenese-1, *VDD* vitamin D deficiency (N = 5).

VDD did not modify the expression of the *CBS* and *CSE* genes measured as mRNA (Fig. 5a, c). However, the protein expression of CBS (*P* = 0.04) and CSE (*P* = 0.015) was significantly reduced (Fig. 5b, d). Opposite, VDD markedly up-regulated testicular HO-1 both at the mRNA (*P* = 0.04) and protein (*P* = 0.011) levels compared to the controls (Fig. 5e, f). Consistent with western blot, the immunofluorescence findings showed low immunoreactivity of CBS and CSE in the testicular germ cells and spermatozoa in VDD-treated mice relative to the controls, while strong immunoreactivity was observed for HO-1 in the VDD group in comparison with the control group (Fig. 5g–i).

## Discussion

We tested the effects of vitamin D deprivation on the male reproductive function in a mice model. Our model effectively induced a substantial vitamin D deficiency without causing significant disturbances in calcium metabolism, despite the crucial role of vitamin D in intestinal calcium absorption. It was anticipated that the reduced vitamin D concentration would result in hypocalcemia and subsequent hyperparathyroidism^17^. Therefore, the lack of hypocalcemia observed in our mice with reduced vitamin D levels is intriguing and represents a unique aspect of our model, which closely aligns with the human condition. The prevalence of vitamin D deficiency without accompanying hypocalcemia in humans is noteworthy. It is plausible that any decrease in calcium absorption caused by vitamin D deficiency is counterbalanced by the supplementation of higher quantities of calcium and phosphates, similar to individuals consuming substantial amounts of dairy or other food sources rich in these minerals.

Indeed, it was already shown that the calcium deficiency developed by VDD mice can be entirely corrected by calcium supplementation^35^ and our results further endorse those findings. The mice under VDD exerted increased body weight, which fits with the already known obesogenic potential of vitamin D deprivation^36–38^. This is in contrast with Fu et al.^39^ which reported no significant difference in body weight between VDD mice and their controls. Differences in the experimental design, vitamin D-deficient diets, and mouse strains may explain this discrepancy. In any case, our data point to some calcium-independent effects of vitamin D shortage on lipid metabolism.

VDD mice had impaired spermatogenesis associated with signs of oxidative damage and decreased protein levels of the transsulfuration enzymes CBS and CSE whereas both the expression and the protein of the enzyme HO-1 were increased. The impairment of sperm concentration, motility, and morphology from the VDD diet that we recorded is consistent with previous findings in mice^39–41^, rats^42^, and humans^16,43^. Shahreza et al.^41^ reported a significant decrease in sperm motility after 10 weeks of VDD in mice. Similarly, in VDR-null mice^35^, and in mice with 1α—hydroxylase deletion^44^, resulting in VDD, histological abnormalities of the testis, reduced sperm concentration and motility and enhanced sperm abnormal morphology were observed. This is confirmed by in vitro studies showing that adequate levels of vitamin D are necessary for human sperm^45,46^. Finally, vitamin D regulates cholesterol outflows of human sperm, influences sperm protein serine and threonine phosphorylation, and thus improves sperm survival ability^47^.

The most evident explanation for the impaired spermatogenesis in our study is the deep suppression of testosterone following VDD with vitamin-deficient animals having just 2% of the testosterone circulating in the controls. This is a new and somehow surprising finding. In humans, it was shown that vitamin D supplementation was effective in increasing (already normal) testosterone levels^48^ but a following study found no association between vitamin D status and testosterone in young men^49^. However, we showed that in mice the sperm defects were more pronounced in animals with a deeper shortage of vitamin^40^, which was not the case of the above clinical studies, i.e., a deep shortage may be necessary to see effects on testosterone.

Most of the enzymes involved in steroidogenesis have vitamin D response element (VDRE) in their promoters and may indeed suffer low vitamin levels. In a recent study in mice a VDR knock-out model, supposed to abolish the VDR-mediated genomic effects of vitamin D, resulted in a suppression of just 30% of circulating testosterone^50^, which is a lot milder deficit compared to our finding. However, besides genomic effects based on interaction with the nuclear receptor (VDRn), vitamin D is known to exert non-genomic effects likely dependent on another vitamin D binding molecule in plasma membranes, VDRm^51^. The nuclear and the membrane ligands are different proteins^52^, thus coded by different genes so that the previously reported VDR knock-out model could not block the non-genomic and VDRm-dependent effects. Therefore, VDR knock-out animals still benefit from the non-genomic effects of vitamin D that are instead as well inhibited if the vitamin is not available, which is the case in our model. On this basis, we speculate that the effects of vitamin D on testosterone release are at least in part non-genomic and that these effects occur only in case of deep vitamin deprivation.

Our study on VDD animals revealed notable elevations in sperm ROS, lipid peroxidation, DNA damage, and apoptosis of spermatogenic cells. Prior research has consistently indicated the involvement of vitamin D in apoptosis, with vitamin D deficiency leading to reduced sperm count and motility. These effects are attributed to the diminished proliferation of spermatogenic cells and heightened apoptosis within the same cell population^39,44^. These findings collectively indicate clear evidence of ongoing oxidative damage and disrupted redox metabolism.

OS can impair spermatogenesis with a variety of mechanisms and may explain our findings, however, we need to understand how VDD triggers OS and testosterone decrease. Low testosterone was shown to trigger OS in mice independently of VDD^53^, which fits with our findings, however, a direct effect of VDD on OS was likely in place. The expression of the *CBS* gene, a master regulator of redox metabolism, is under the control of VDRE so in vitamin D deprivation a lower expression of the gene was expected with decreased expression also of the *CSE* gene due to lower availability of its substrate. However, in VDD mice we found unchanged levels of the CBS and CSE mRNA whereas it was their protein level to be significantly affected. Thus, post-transcription modifications and/or faster protein catabolism are likely in place, and again, VDD was not acting at the genomic level. On the other side, whatever the mechanism, a shortage of CBS enzymatic activity may explain the reduced testosterone secretion. It has been shown that the CBS product H_2_S regulates testosterone synthesis by inhibiting phosphodiesterase (PDE) expression via sulfhydryl modification and activating cAMP/PKA pathway^54^.

CBS releases H_2_S when working in its alternative modality, which was shown to be activated by CO binding to the heme center of CBS following HO-1 activation by endoplasmic reticulum (ER) stress^27^. Accordingly, in VDD we found increased expression of HO-1 both at the genetic and protein level, which we interpret as the activation of a compensatory, mechanism. VDD has been shown to induce ER stress and vitamin D supplementation to correct it in mice macrophages^55^ and in rats’ brain^56^, just to mention some evidence and ER stress was likely developing also in our deprived animals. We speculate that such ER stress-activated HO-1 with the aim to trigger corrective actions including H_2_S release.

VDD mice also suffered impaired sperm chromatin maturation with excessive retention of histone proteins and defective protamination. Sperm chromatin maturation is strictly dependent on extensive methylation of DNA and histones^57^, which again points to the one-carbon metabolism: A lack of Hcy re-methylation may cause a deficit of S-adenosyl-methionine and decreased feed to transmethylations paralleled by an accumulation of Hcy. We found indeed increased serum Hcy in our VDD mice. However, we also recorded a huge increase in vitamin B12, which is as well necessary for Hcy re-methylation and is usually decreased in hyperhomocysteinemia. However, the B12 form involved in Hcy re-methylation is methylcobalamin and the activation of B12 to the methylated form is redox dependent, likely mediated by an action of H_2_S released from CBS and CSE^58^. We speculate that B12 accumulated because of reduced conversion to methylcobalamin due to lack of H_2_S, resulting in increased Hcy and deranged methylations leading to sperm chromatin impairment.

A main limitation of the present study is that we did not check our animals for GSH and H_2_S release, therefore we cannot definitely confirm our interpretation. Moreover, our animals suffered very deep vitamin D deprivation leading to extreme testosterone shortage and both these conditions are unlikely to occur in the clinical setting. However, the prevalence of severe clinical vitamin D deficiency is reported to be in double digit frequency and less severe deficits, possibly leading to subtler and undiagnosed clinical consequences, are quite common^60^. Our data further endorse the need for close monitoring, and in case correction, of vitamin D status in infertile men. Moreover, assumed the strong negative effect of the deficiency on testosterone level that we report, the vitamin D status should be included also in the work-up of men with low testosterone level independently of infertility.

## Conclusion

In summary, we used a VDD model in mice to demonstrate impaired spermatogenesis due to OS and defective chromatin maturation. A deep reduction in circulating testosterone appeared as a main connection between VDD and sperm defects in our animals. Our data point to the non-genomic and VDRn-independent effects of vitamin D in this context and to a deficit of H_2_S release as a primary effector of the damage in our model, which warrants for further confirmation in duly designed experimental models. Meantime, we call for the need to check the vitamin D status in male infertility and subclinical male hypogonadism.

## Materials and methods

### Animals and study design

This study was approved by the Scientific Ethics Committee of Royan Institute (IR.ACECR.ROYAN.REC.1400.142), and we confirm that all the methods and protocols were carried out according to ARRIVE guidelines and regulations^60^ and all methods were performed in accordance with the relevant guidelines and regulations. Sixteen healthy male C57 mice (11–13 g, 4 weeks old) were involved in this study. The mice were kept in the animal house of the Royan Institute for Animal Biotechnology (Isfahan, Iran). All animals were permitted free access to food and water and maintained at 22 ± 2 °C, 45–65% humidity, and a day/night 12 h/12 h photoperiod. After one week acclimatization, mice were randomly divided into two groups (N = 8) as follows:

Group 1: (Control group) received a standard chow diet (Standard AIN93G Rodent diet).

Group 2: (Vitamin D deficient (VDD) group) received a customized vitamin D deficient diet (modified AIN-93G diet, 0 IU/kg of vitamin D3, 0.52% Calcium, and 0.23% Phosphorous). All mice were overfed with calcium and phosphorus. After 14 weeks all the animals were sacrificed. This study is the continuation of the study by Pouriayevali et al.^40^ (IR. ACECR. ROYAN. REC.1399.122).

### Samples collection and assays

At the end of the 14th week, all animals were euthanized by intraperitoneal injection of a cocktail of Ketamine (90 mg/kg body weight) and Xylazine (10 mg/kg body weight) using American Veterinary Medical Association​ (AVMA) Guidelines for the Euthanasia of Animals (https://www.avma.org) and the final body weight and testicular weight of mice were assessed. Blood samples were acquired using cardiac puncture, transferred to 5 ml vials, and centrifuged at 2500 rpm for 15 min, then the serum portion was separated and frozen at − 20 °C until analysis. The levels of serum testosterone (Testosterone II, Roche, Switzerland), vitamin D3 (Vitamin D total II, Roche, Switzerland), calcium (Calcium C311, CAP, America), folate (Folate III, Roche, Switzerland), and B12 (Vitamin B12 II, Roche, Switzerland) were analyzed using ELISA kits (Elecsys 2010 and Cobas e411 analyzers) according to manufacturer’s protocol and absorbance was measured by spectrophotometry at 450 nm. The serum Hcy concentration was measured by high-performance liquid chromatography (HPLC). After separating the epididymis and washing it in PBS at 37 °C, the cauda of the epididymis was incubated in the sperm-washing medium (VitaSperm, Inoclon, Iran) for 30 min at 37 °C to prepare sperm suspension. To decrease observational variances, all parameters were evaluated by a single blinded investigator.

### Testicular histopathological assay

The left testis from animals was fixed using the 10% buffered formalin solution overnight. Next, formalin-fixed testes were embedded in paraffin blocks. Paraffinized specimens were cut (5 μm-thick) with a microtome and mounted on glass slides. Next, testicular tissue sections were stained with Hematoxylin and Eosin (H&E) for morphological analysis. Morphological assessment of the testes was carried out with a light microscopic system (CX31 OLYMPUS, Japan) at × 400 magnification^61^.

### Sperm analysis

A sperm counting chamber (Makler) was employed for sperm concentration, which was displayed as millions of spermatozoa/ml. Five μl of extracted epididymal spermatozoa were placed in a sperm counting chamber. For each specimen, sperm heads were counted in 5 rows, then counted sperms were divided by 5. For the percentage of sperm motility, 5 μl of the sperm suspensions were located on a pre-warmed slide, and then the motility of 200 spermatozoa was evaluated through visual inspection of the movement types under a light microscope (CX31 OLYMPUS, Japan; × 400 magnification). Finally, the sperm motility percentage was calculated by the formula:

Sperm motility (%) = [number of motile sperm (progressive + non-progressive)/total sperm] × 100. Total sperm motility and progressive motility were recorded.

To evaluate sperm morphology, 20 μl of washed spermatozoa were stained with 40 μl Eosin (Merck, Darmstadt, Germany). After 5 min, 60 µl of Nigrosine (Merck, Darmstadt, Germany) was append to this mixture. Then, smears were prepared with 20 μl of the stained samples. For each sample, 200 spermatozoa were randomly assessed under × 1000 magnification (CX31 OLYMPUS, Japan)^62^.

### Sperm chromatin maturation assays

For each sperm sample, we evaluated the sperm chromatin maturity using Aniline blue (AB) staining. In brief, 20 μl of washed epididymal spermatozoa were smeared and air-dried at room temperature, then fixed for 2 h in 3% glutaraldehyde in 0.2 M phosphate buffer (pH 7.2). Slides were stained with aqueous AB (5%) in 4% acetic acid (pH 3.5) for 120 min and then washed with PBS. For each slide, 200 sperm cells were randomly counted under the light microscope (× 1000 magnification, CX31 OLYMPUS, Japan), and spermatozoa with unstained nuclei were considered normal (AB−) while those dark blue stained were considered abnormal (AB +)^63^.

Histone replacement by protamine occurs during spermiogenesis. Chromomycin A3 (CMA3) was utilized to assess the sperm protamine levels. For this evaluation, twenty μl of washed epididymal spermatozoa were fixed with methanol: acetic acid (3:1, v/v, Merck, Darmstadt, Germany) for 5 min at − 4 °C. Then, 20 μl of fixed sperm sample was smeared on the slide and allowed to dry at room temperature. Next, smears were stained with 100 µl of 0.25 mg/ml CMA3 solution (Sigma Chemical Co., St Louis, USA) for 1 h. After, each slide was washed with PBS (twice), air‐dried, and coated with a coverslip. on each slide, 200 spermatozoa were evaluated by a fluorescent microscope (BX51 OLYMPUS, Japan, × 1000 magnification). Spermatozoa that stained as bright yellow (CMA3 ^+^ with deficient protamine) were reported^63^.

### Evaluation of sperm DNA damage

Sperm DNA damage was assessed by Acridine orange (AO) staining^64^, which is a fluorescent dye. Briefly, washed spermatozoa were smeared on a slide and air-dried. Then slides were fixed with Carnoy’s solution (methanol:acetic acid 3:1, v/v, Merck, Darmstadt, Germany) at 4 °C for 2 h. Next, the slides were washed with PBS and stained with AO stain that was freshly prepared in citrate–phosphate buffer (80 ml 0.1 M citric acid + 5 ml 0.3 M NaH_2_PO_4_, pH 2.5) for 90 min. After, the slides were washed with PBS. At least 200 spermatozoa were evaluated for each sample by a fluorescent microscope (BX51 OLYMPUS, Japan, × 1000 magnification) and DNA of sperms with green fluorescence was assumed as normal double‐stranded whereas DNA showing an orange/red fluorescence was considered as with abnormal denatured DNA. The sperm DNA damage percentage was reported for each sample.

### Apoptosis assessment

Apoptosis was evaluated using a TUNEL kit, following the manufacturer's instructions. Histological sections measuring 5–6 μm were de-paraffinized and rehydrated. Next, the sections were treated with 1 μl of proteinase K (15 μg/ml in 10 mM Tris/HCl, pH: 7.4) for 30 min. The slides were then washed with a Phosphate-buffered saline (PBS) solution. Afterward, the slides were exposed to 25 μl of TUNEL solution, which consisted of a mixture of 50 μl enzyme solution and 450 μl label solution, for 60 min. Following this, the slides were covered with 25 μl of POD-convertor and incubated for 30 min. Subsequently, 25 μl of DAB substrate, composed of 1 μl of DAB and 10 μl of DAB substrate, was added to the slides and incubated for 60 s. Finally, the sections were counterstained with hematoxylin. The number of apoptotic germ cells per seminiferous tubule was then counted and compared between the groups using the same criteria^65^.

### Assessment of intracellular ROS production and lipid peroxidation

Intracellular H_2_O_2_ level in the caudal epididymal spermatozoa was assessed by Dichloro-dihydro-fluorescein diacetate (DCFH-DA) assay. DCFH-DA is a cell-permeable molecule, that is hydrolyzed to DCFH inside cytosol where it can be oxidized by ROS, to generate 2,7-dichlorodihydrofluorescein (DCF), which can be monitored by fluorescence-based techniques. One million spermatozoa in PBS were incubated with 0.5 μM DCFH-DA at 37 °C for 30 min in a dark condition. Then, sperm H_2_O_2_ levels were assessed using a FACSCalibur flow cytometer (Becton Dickinson, San Jose, CA, USA). The percentage of sperm cells with intracellular H_2_O_2_ was reported for each sample^66^.

We assessed sperm membrane lipid peroxidation according to Aitken et al.^67^. In brief, sperm samples (2 × 10^6^ cells) were incubated with the final concentration of 5 µM BODIPY C11 for 30 min at 37 °C. Then, samples were washed with PBS (500 g for 5 min), and the sperm lipid peroxidation percentage for each sample was assessed using a FACSCalibur flow cytometer (Becton Dickinson, SanJose, CA, USA). In addition, positive controls were attained after the addition of 2 mM H_2_O_2_ to sperm suspensions.

### RNA isolation and qRT-PCR protocols

Fifty mg of testicular tissue was homogenized with 1 ml of TRIzol reagent (Yekta Tajhiz Azma, Iran). The RNA concentration and purity were evaluated by reading the absorbance at 230 nm, 260 nm, and 280 nm with a NanoDrop1000 spectrophotometer. The cDNA was synthesized from total RNA by cDNA synthesis kit according to the manufacturer’s instruction (Biotechrabbit™ cDNA Synthesis Kit). Subsequent Real-time PCR was executed with SYBR green (Yekta Tajhiz Azma, Iran) fluorescent dye using ABI (Applied Biosystems StepOnePlus™). All data were normalized to glyceraldehyde-3-phosphate dehydrogenase (GAPDH) and expressed as 2^−ΔΔCt^^68^. The list of primers is revealed in Table 1.

**Table 1:** Primer sequences of genes analyzed by real-time PCR.

Name	Forward (5′–3′)	Reverse (5′–3′)	Tm (°C)
Cystathionine-β-synthase (CBS)	GGGCGAAGTGGTCCATCTC	GTTGGCAAAGTCATCTACAAGCA	56
Cystathionine-γ-lyase (CSE)	GACTCTACATGTCCGAATGG	AACCTGTACACTGACGCTTCA	56
Heme oxygenase-1 (HO-1)	ATGTTGACTGACCACGAC	GCCCCACTTTGTTAGGAAA	56
Glyceraldehyde-3-Phosphate Dehydrogenase (GAPDH)	TGCCGCCTGGAGAAACC	TGAAGTCGCAGGAGACAACC	60

Tm: primer melting temperature.

### Western blotting

Briefly, protein extraction from the testicular tissue was performed using the TRIzol reagent (Yekta Tajhiz Azma, Iran)^62^. The protein concentration in each sample was estimated by Bradford’s assay. For all samples, 30 μg protein was loaded onto 10% sodium dodecyl sulfate polyacrylamide gels (SDS-PAGE) and then transferred to polyvinylidene difluoride (PVDF) membranes. The membranes were blocked in PBS containing 10% skimmed milk powder and incubated overnight, and then rinsed in PBS and probed with the primary antibodies including anti-CBS monoclonal (CBS (B-4): sc-133154, 1:400), anti-CSE monoclonal (CTH (F-1): sc-374249, 1:200), and anti-HO-1 monoclonal (Heme Oxygenase 1(F-4): sc-390991, 1:1000). Afterward, the membranes were washed (three-time, 15 min) in PBS and incubated for 1 h at room temperature with horseradish peroxidase (HRP) conjugated anti-mouse IgG (Dako, P0447, 1:5000) for anti-CBS, anti-CSE, anti-HO-1, and β-actin antibodies. Finally, the presence of the protein bands was identified by an enhanced chemiluminescence system (ECL, Santa Cruz, USA) following the manufacturer’s instructions. To reduce costs, membranes were cut into two or three pieces before exposure to the first antibody or the specific antibody. Regarding the repeat, each blot represents at least 4 to 5 samples and they are not repeats of one sample. Data normalization was performed using β-actin as an internal control, and the signal quantification was achieved by Image J software.

### Immunofluorescence of mouse testicular tissue

The fixed mouse testes were surrounded in paraffin wax and then sections were cut (5 μm-thick). After deparaffinization and rehydration, a preheated sodium citrate buffer (pH 6.0) was used for antigen retrieval for 10 min. Thereafter, the sections were permeabilized with 0.3% Triton X-100 for 30 min and blocked with 10% normal goat serum (NGS) for 30 min at 37 °C. Subsequently, they were exposed to primary antibodies: anti-CBS monoclonal (CBS (B-4): sc-133154, 1:50), anti-CSE monoclonal (CTH (F-1): sc-374249, 1:50), and anti-HO-1 monoclonal (Heme Oxygenase 1(F-4): sc-390991, 1:50) overnight at 4 °C. Next, slides were incubated with the secondary antibody anti-mouse IgG-FITC (1:400, Chemichon, AP124F) and 4′,6-diamidino-2-phenylindole (DAPI) or propodeum iodide (PI). Subsequently, the slides were washed, and mounted with anti-fade glue (Gold antifade reagent; Molecular probes; USA; Cat. No. P36934). Then, the samples were evaluated by a fluorescent microscope (BX51 OLYMPUS, Japan)^33^.

### Statistical analysis

The normality of data was checked by the Shapiro–Wilk test. All results were presented in mean ± standard deviation (SD), and independent-sample t-tests were applied to determine differences between groups by SPSS software (SPSS Science, Chicago, IL, USA). A *P* value < 0.05 was considered statistically significant. GraphPad Prism statistical software package version 8 (GraphPad Software) was used for graph design.

### Supplementary Information

Supplementary Information.

## Data Availability

All data generated or analyzed during the current study available from the corresponding author on reasonable request.
