# Supplementary material for: The role of the transsulfuration pathway in spermatogenesis of vitamin D deficient mice
Source: Sci Rep. 2023 Nov 6;13:19173. doi: 10.1038/s41598-023-45986-4 (PMC10628119; doi:10.1038/s41598-023-45986-4)
Supplement: Supplementary file 1 — Supplementary Information. [file 41598_2023_45986_MOESM1_ESM.docx]

Thank you for your comment. In accordance with digital image integrity and standards, we have not manipulated the Electrophoretic gels of the Western blot. We have included the original, unprocessed images in the supplementary file.

**VDD**

**Control**


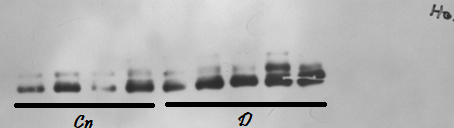

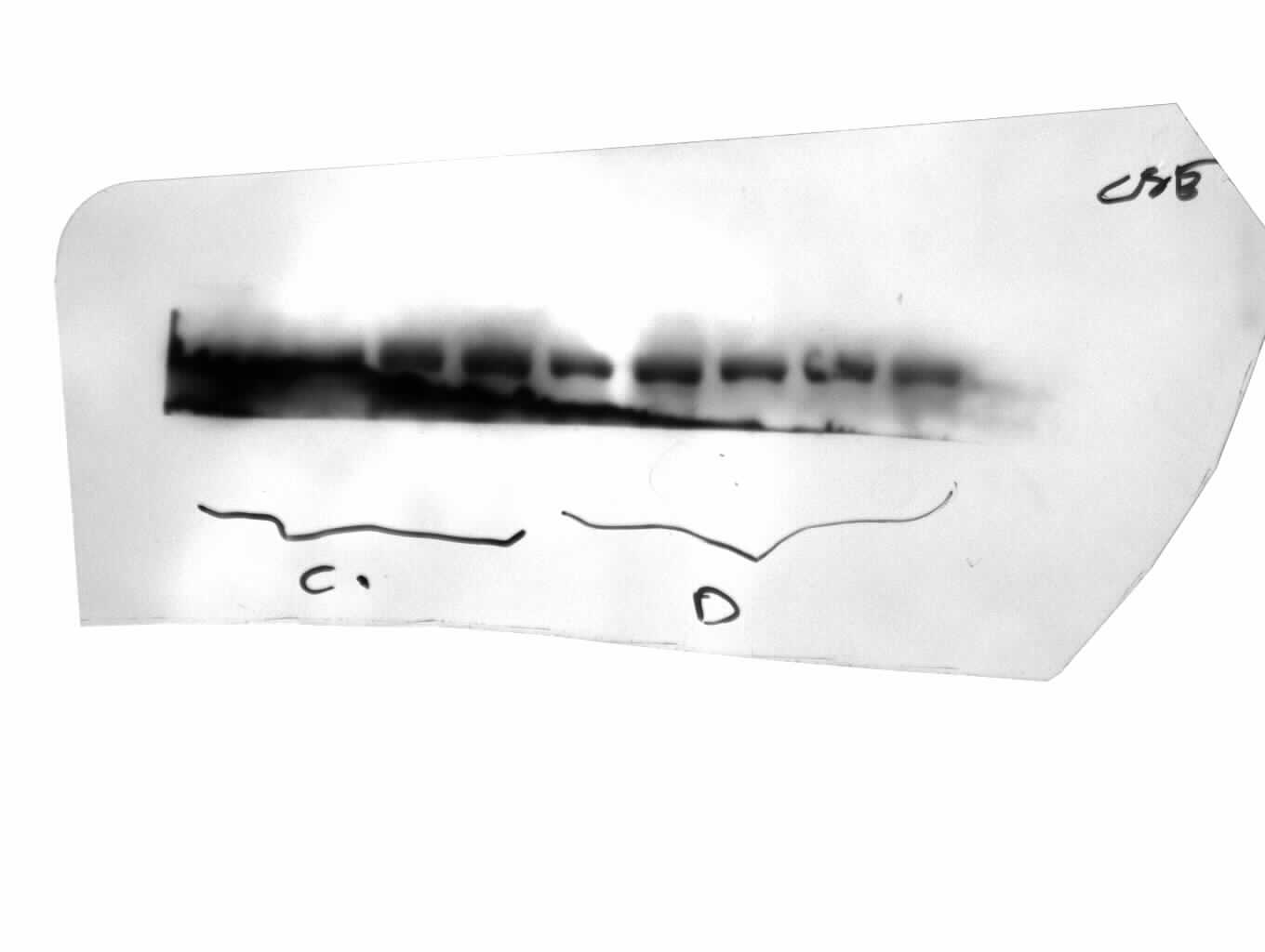

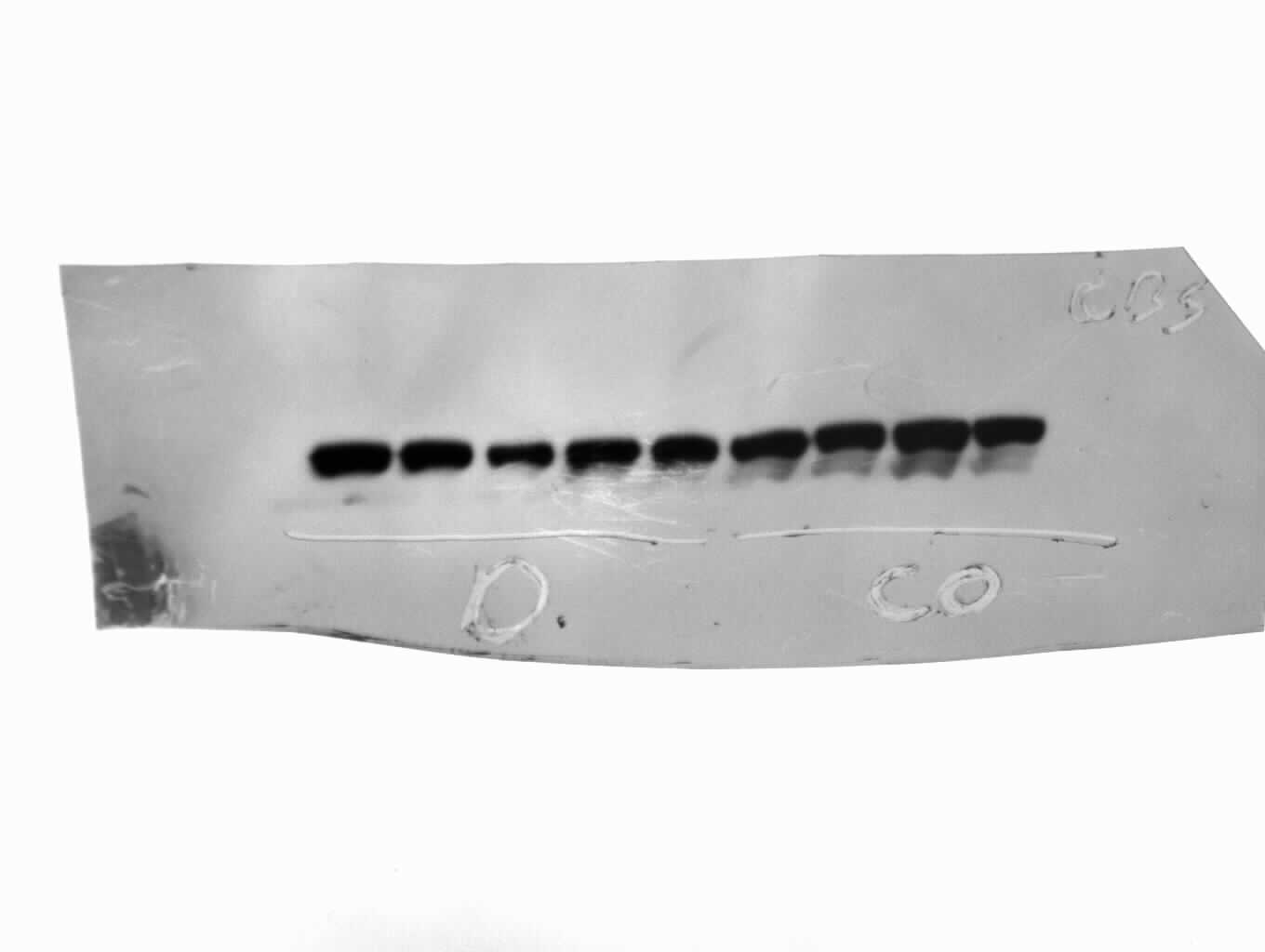


**Control**

**HO-1**

**VDD**

**CSE**

**VDD**

**Control**

**CBS**


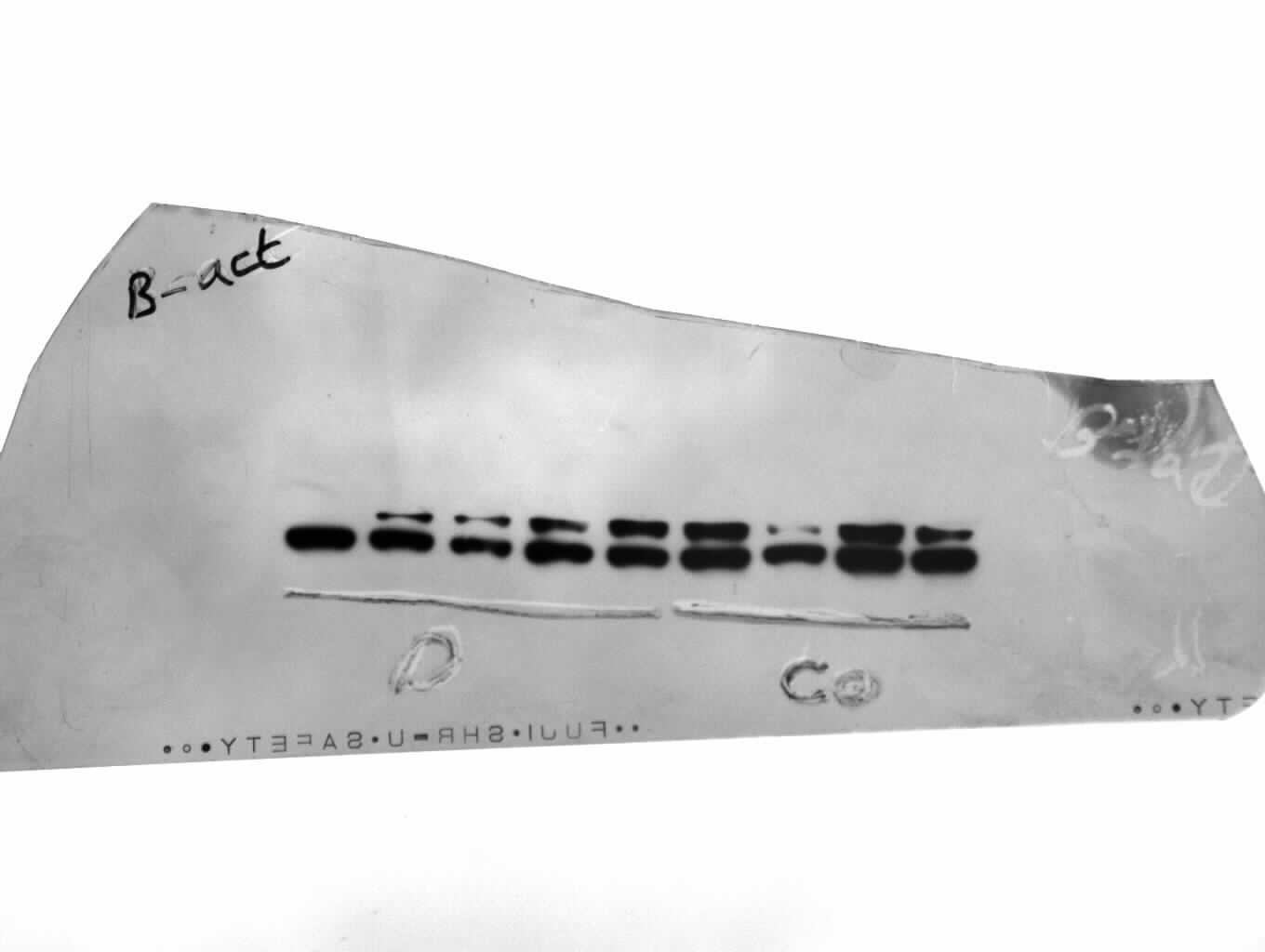


**Β-actin**

**VDD**

**Control**
